# Effects of butter naturally enriched with conjugated linoleic acid and vaccenic acid on blood lipids and LDL particle size in growing pigs

**DOI:** 10.1186/1476-511X-7-31

**Published:** 2008-08-29

**Authors:** Anna Haug, Per Sjøgren, Nina Hølland, Hanne Müller, Nils P Kjos, Ole Taugbøl, Nina Fjerdingby, Anne S Biong, Eirik Selmer-Olsen, Odd M Harstad

**Affiliations:** 1 Dept of Animal and Aquacultural Sciences, Norwegian University of Life Sciences, Aas, Norway; 2 King Gustaf V Research Institute, Karolinska Institute, Stockholm, Sweden; 3 Norwegian School of Veterinary Science, Oslo, Norway; 4 Tine BA, Oslo, Norway

## Abstract

**Background:**

Cow milk is a natural source of the cis 9, trans 11 isomer of conjugated linoleic acid (c9,t11-CLA) and trans vaccenic acid (VA). These fatty acids may be considered as functional foods, and the concentration in milk can be increased by e.g. sunflower oil supplementation to the dairy cow feed.

The objective of this study was to compare the effects of regular butter with a special butter naturally enriched in c9,t11-CLA and VA on plasma lipids in female growing pigs. The experimental period lasted for three weeks and the two diets provided daily either 5.0 g c9,t11-CLA plus 15.1 g VA or 1.3 g c9,t11-CLA plus 3.6 g VA.

**Results:**

The serum concentrations of c9,t11-CLA, VA and alpha-linolenic acid were increased and myristic (14:0) and palmitic acid (16:0) were reduced in the pigs fed the CLA+VA-rich butter-diet compared to regular butter, but no differences in plasma concentrations of triacylglycerol, cholesterol, HDL-cholesterol, LDL-cholesterol, LDL particle size distribution or total cholesterol/HDL cholesterol were observed among the two dietary treatment groups.

**Conclusion:**

Growing pigs fed diets containing butter naturally enriched in about 20 g c9,t11-CLA plus VA daily for three weeks, had increased serum concentrations of alpha-linolenic acid and decreased myristic and palmitic acid compared to pigs fed regular butter, implying a potential benefit of the CLA+VA butter on serum fatty acid composition. Butter enriched in CLA+VA does not appear to have significant effect on the plasma lipoprotein profile in pigs.

## Background

Milk and dairy products have long traditions in human nutrition, but for some decades milk fat has been associated with negative health effects. However, the association between milk fat and plasma lipids is ambiguous, and a paradox. Several studies show no convincing evidence that dairy products increase the risk of cardiovascular disease and that milk is harmful [1-4]. Some studies indicate that a moderate intake of milk fat may reduce the risk of cardiac diseases, possibly through reduced formation of small dense low density lipoprotein particles (sdLDL) [5]. The sdLDL are thought to undergo oxidation more readily, or to be harder bound to the arterial endothelia surface [6]. Dairy milk fat contains numerous fatty acids that might affect formation of sdLDL, such as saturated fatty acids, c9,t11-CLA isomer and VA [7,8]. Evidence for hypolipidemic properties of c9,t11-CLA has been given, and administration of CLA has been shown to modulate plasma lipid concentration in both human and animal models, and to reduce markers associated with atherogenic risk [8-10]. These findings have led to considerable interest in methods for naturally increasing the c9,t11-CLA content in milk, and milk products that are naturally enriched in CLA has been advocated. CLA is a group of polyunsaturated fatty acids found naturally in beef, lamb and dairy products, and c9,t11-CLA is the main form of CLA (18:2 c9,t11). It can be produced in ruminants by bacterial isomerisation of linoleic acid (18:2 c9,c12) in the rumen. In ruminants and non-ruminants it can be produced in most tissues by delta-9 desaturation of VA (18:1 t11) [11,12]. The concentration of c9,t11-CLA and VA in milk fat is highly dependent on the feed composition; VA is an intermediate product of biohydrogenation of unsaturated fatty acids in the rumen, and feedstuff rich in linoleic or α-linolenic acids enhance CLA and VA in milk [13]. Since c9,t11-CLA in milk fat is associated with VA, milk rich in c9,t11-CLA is also rich in VA, its precursor [12]. In general, trans fatty acids are associated with increased plasma cholesterol and risk for coronary heart disease. Therefore the concentration of VA in milk has been of concern. However, VA's effect on plasma cholesterol has not been entirely understood since epidemiological studies have shown that trans fatty acids from animal sources did not increase risk for coronary artery disease [14].

The objective of the study presented here was to compare effects on plasma lipid- and fatty acid profile in growing pigs that had been given diets containing regular butter (REG) or butter naturally enriched in CLA plus VA.

## Results and discussion

### Feed intake and weight gain

In the present study, there were no significant differences in the feed intake (in average 1.65 kg and 1.67 kg per day), weight gain (17.9 kg and 18.4 kg) and final body weight (61.2 kg and 61.9 kg) in the REG and CLA+VA treatment groups, respectively (data not shown). The CLA isomer involved in decreasing body fat is the t10,c12-CLA isomer, but there are no concluding evidence of such effects of the c9,t11-CLA isomer [15,16].

### Fatty acid (FA) composition of diets

The CLA+VA rich butter diet differed from the regular butter diet with a higher concentrations of c9,t11-CLA, VA, oleic acid (18:1,9c), linoleic (18:2) and α-linolenic acid (18:3), and less 10:0, 12:0, myristic acid and palmitic acid (Table 1). This diet provided daily 5.0 g CLA and 15.1 g VA, and the intake of these two fatty acids was about four times higher in the CLA+VA dietary treatment compared to the REG dietary treatment. Intake of CLA+VA as percent of average body weight was about 0.04% in the CLA+VA treatment group, and 0.01% in the REG group. Alpha-linolenic acid was about 30% higher and palmitic and myristic acid about 25% lower in the CLA+VA diet compared to REG.

**Table 1:** Fatty acid composition of the diets, (g/100 g fatty acid methyl ester).

Fatty acid	REG^a^	CLA+VA^b^
10:0	2.43	1.61
12:0	2.88	2.13
14:0	10.32	8.02
14:1	0.93	0.79
16:0	28.09	20.84
16:1	1.65	1.13
18:0	11.09	10.75
18:1 t11	1.36	5.62
18:1 9c	22.03	25.11
18:2 n-6	7.09	9.21
18:3 n-3	1.13	1.51
c9,t11-CLA	0.53	1.85
20:4 n-6	0.07	0.09
20:5 n-3	0.07	0.10
22:5 n-3	0.07	0.07
22:6 n-3	0.02	0.05
		
Sum^c^	89.80	88.88

^a^Diet with regular butter as fat supplement

^b^Diet with CLA+VA rich butter as fat supplement

^c ^Butter contains several unidentified fatty acids in small amounts (areal percent less than 0.5%).

To provide a high intake of natural CLA and VA, the experimental diets were rich in fat; 19% fat in both diets, giving as much as 46% of the energy (E%) from fat. The pigs liked the diets, and in accordance to others [17] pigs tolerated well a high fat diet.

### Serum fatty acids

At the start of the experiment there were no differences between the two experimental groups in the concentration of different FA in serum (data not shown). In contrast, at the end of the study it was significant differences between the diet groups in concentration of several serum FA (Table 2).

**Table 2:** Overall means in serum fatty acid concentration (g/100 g fatty acid methyl ester)^a^

Fatty acid	REG^b^	CLA+VA^c^	*p*
10:0	0.17 ± 0.05	0.12 ± 0.03	0.08
12:0	0.67 ± 0.44	0.54 ± 0.13	0.52
14:0	1.36 ± 0.31	1.03 ± 0.12	0.04*
14:1	0.32 ± 0.04	0.28 ± 0.05	0.18
16:0	20.1 ± 0.90	17.1 ± 0.75	<0.01*
16:1	1.02 ± 0.17	0.89 ± 0.13	0.16
18:0	16.3 ± 1.6	14.6 ± 0.9	0.054
18:1 9c	16.2 ± 1.7	16.2 ± 0.3	0.98
18:2 n-6	20.0 ± 1.3	21.6 ± 2.4	0.20
18:3 n-3	0.81 ± 0.13	1.02 ± 0.09	0.01*
18:1 t11	0.27 ± 0.05	1.09 ± 0.18	<0.01*
c9,t11-CLA	0.28 ± 0.08	0.80 ± 0.07	<0.01*
20:4 n-6	8.2 ± 1.0	8.1 ± 0.4	0.89
20:5 n-3	1.49 ± 0.19	1.46 ± 0.06	0.65
22:5 n-3	1.41 ± 0.15	1.47 ± 0.19	0.57
22:6 n-3	1.74 ± 0.34	1.46 ± 0.17	0.10

^a^Values given as mean ± SD, and p-values, n = 6.

^b^Diet containing regular butter as fat supplement

^c^Diet containing CLA+VA rich butter as fat supplement

* p < 0.05.

The main FA in serum are palmitic acid, stearic acid, oleic acid and linoleic acid, together accounting for about 70% of total FA (Table 2). Pigs fed the CLA+VA diet had a 3.4 fold higher serum concentrations of c9,t11-CLA plus VA compared to the REG diet group. Alpha-linolenic acid was 25% higher, and myristic and palmitic acid was 25% and 15% lower in the serum of CLA+VA compared to the REG dietary treatment (Table 2). The mirroring effect of dietary FA on serum FA is in accordance to several studies [5,18,19]. The favourable increased serum concentrations of the omega-3 fatty acid α-linolenic acid and the decrease in the saturated palmitic and myristic acids may indicate that the CLA+VA rich butter can have a positive role in the western diet.

### Plasma cholesterol, triacylglycerol and lipoproteins

At the start of the study, no differences between the groups were observed for plasma concentrations of total cholesterol, LDL-cholesterol, high density lipoprotein (HDL)-cholesterol, sdLDL subclass particle diameter, the ratio between total cholesterol and HDL cholesterol, free fatty acids and triacylglycerol.

The dietary fatty acids in the CLA+VA treatment were more favourable than in the REG diet; i.e. more oleic acid (18:1c9), linoleic acid (18:2 n-6), and α-linolenic acid (18:3 n-3), c9,t11-CLA and less myrisitc acid (14:0) and palmitic acid (16:0) [20] (Table 1). In spite of intake of the more favourable fatty acids in the CLA+VA treatment group, the plasma concentration of recognized risk markers for atherosclerosis such as total cholesterol, LDL-cholesterol, sdLDL, the ratio between total cholesterol and HDL cholesterol and triacylglycerol did not decrease, and HDL-cholesterol was not increased (Table 3). The lack of response of CLA+VA enriched butter on lipoprotein profiles is in accordance to a human study [9]. It is also shown no response on plasma lipoproteins in growing pigs fed diets containing unsaturated plant fatty acids compared to saturated animal fat [21]. In the present experiment the two types of butter differed in several fatty acids. Butter contains numerous fatty acids in minor amounts that may have potential bioactive effects on lipid metabolism. The CLA+VA butter contained a high concentration of VA. The intake of VA was considerable in the CLA+VA group; 15.1 g per day compared to 3.6 g per day in REG treatment group. Vaccenic acid is a substrate for c9,t11-CLA in the animals own tissue, and in this way it is desirable [11], but trans fatty acids in general has been attributed to increased plasma cholesterol and also other pathogenetic factors [20,22]. Given the high dosage of VA supplied daily in the CLA+VA diet, it can not be excluded that VA might have had opposite effects on plasma lipoproteins than CLA and other desirable fatty acids. It has been reported by others that the natural combination of CLA and VA had no detrimental effect on the blood lipid profile in humans [9]. The fact that the high intake (about 20 g per day for pigs weighing in average about 50 kg) of CLA+VA in combination improved serum fatty acid profile and did not have an unfavorable effect of plasma lipoproteins can stimulate to new strategies to develop natural CLA+VA rich dairy products.

**Table 3:** Plasma lipids, lipoproteins and percent distribution of LDL-particles LDL-I, LDL-II, LDL-III and LDL-IV^a^

	REG^b^	CLA+VA^c^
Triglyceride	0.43 ± 0.07	0.46 ± 0.13
Total cholesterol mmol/l	3.61 ± 0.5	3.50 ± 0.4
HDL cholesterol mmol/l	1.44 ± 0.17	1.32 ± 0.21
LDL cholesterol (calculated)	1.98 ± 0.37	1.97 ± 0.33
Total cholesterol/HDL cholesterol	2.51 ± 0.15	2.65 ± 0.28
LDL cholesterol/HDL cholesterol	1.38 ± 0.15	1.49 ± 0.28
LDL peak size (Å)	243 ± 3.6	243 ± 3.2
Percent distribution^d^		
LDL-I (%)	21.7 ± 7.1	25.0 ± 4.0
LDL-II (%)	47.5 ± 3.4	44.9 ± 1.9
LDL-III (%)	20.5 ± 4.2	18.5 ± 3.3
LDL-IV (%)	8.0 ± 1.8	9.2 ± 1.7
Nonesterified fatty acids mmol/l	0.48 ± 0.18	0.41 ± 0.09

^a^Values given as mean ± SD, n = 6.

^b^Diet containing regular butter as fat supplement

^c^Diet containing CLA+VA rich butter as fat supplement

^d ^Represents the proportions of LDL-I, 27.0–25.0 nm; LDL-II, 25.0–23.5 nm; LDL-III, 23.5–22.5 nm; LDL-IV, 22.5–21.0 nm.

Milk products have been shown to have an apparently beneficial effect on LDL particle size distribution (giving less of the sdLDL) [5]. From the Framingham Offspring Study [23] it has been shown that subjects with a high intake of certain saturated fatty acids (4:0–10:0 and myristic acid) abundantly found in milk products, have lowered levels of sdLDL. Other has shown that saturated fatty acids (especially myristic- and palmitic acid) may affect the distribution of the LDL particles, giving more of the large LDLs [24]. The REG diet contained more 10:0, myrisitic and palmitic acid, but no improvement in LDL particle size was observed in the REG diet group.

## Conclusion

A diet containing natural CLA+VA enriched butter resulted in increased serum concentrations of CLA, vaccenic acid and α-linolenic acid, and reduced concentration of myristic and palmitic acid in pigs compared to a diet containing regular butter, indicating a potential health benefit of the CLA+VA rich butter. However, no differences in plasma lipoproteins and LDL particle sizes were observed among the two dietary treatment groups following three weeks feeding. It is worth noting that a relatively high intake of the trans fatty acid VA did not result in a detrimental effect on the lipoprotein profile when it was in combination with c9,t11-CLA. Perhaps is the combination of fatty acids in milk fat one reason to the milk fat paradox?

## Methods

### Animal care

The experimental research on animals followed internationally recognized guidelines. All animals were cared for according to laws and regulations controlling experiments with live animals in Norway (The Animal Protection Act of December 20th, 1974, and the Animal Protection Ordinance Concerning Experiments with Animals of January 15th, 1996); according to the rules given by Norwegian Animal Research Authority.

### Animals and diets

Twelve growing female pigs (initial weight were 43.4 ± 1.5 kg) of a commercial Norwegian crossbreed ((Landrace × Yorkshire) × (Landrace × Duroc)) were selected for the study. The pigs were reared indoors, and fed two times per day in accordance to NRC requirements for nutrients for growing pigs [25]. A veterinarian examined the pigs every week.

Feed ingredients and fatty acid composition of the two experimental diets are shown in Table 1 and 4, respectively. The dietary treatment (diets produced at Borgen Aktiemolle, Andebu, Norway) was identical except for fat source; regular butter (Tine butter, Oslo, Norway) or CLA+VA rich butter (Table 4). The CLA+VA butter was specially produced for this experiment from milk from dairy cows that were receiving a cereal based commercial feed concentrate (8 kg per day), grass dominated pasture with white clover and supplemented with sunflower oil (500 ml per day, containing 60% 18:2, Karlshamn AB, Karlshamn, Sweden). The CLA+VA rich butter contained 2.1 g c9,t11-CLA and and 6.3 g VA per 100 g fat. The regular butter contained 0.6 g c9,t11-CLA and 1.5 g VA per 100 g fat. The average daily intake of c9,t11-CLA and VA in the CLA+VA treatment group was 5.0 g c9,t11-CLA, 15.1 g VA and in the REG group: 1.3 g c9,t11-CLA, 3.6 g VA.

**Table 4:** Composition of experimental diets, (g per 100 g dry feed).

	REG^a^	CLA+VA^b^
*Ingredients*		
		
Regular butter-fat	16.16	-
CLA rich butter-fat	-	16.16
Oat	6.04	6.04
Wheat bran	39.7	39.7
Soybean meal	19.15	19.15
Rapeseed meal	3.97	3.97
Sugarbeet pulp	10.3	10.3
Ground limestone	1.36	1.36
Monocalciumphosphate	1.20	1.20
Sodium chloride	0.46	0.46
SoftAcid	0.88	0.88
Microminerals, swine^c^	0.08	0.08
L-lysine	0.27	0.27
DL-methionine	0.16	0.16
L-threonine	0.115	0.115
D-cholinechloride	0.063	0.063
Vitaminpremix swine^d^	0.053	0.053

^a^Diet with regular butter as fat supplement

^b^Diet with a special CLA+VA rich butter as fat supplement

^c^Vitamins,: Providing the following amounts per kg of feed: Vitamin A 9000 IU; Vitamin D_3 _1100 IU; Vitamin E 110 mg; Vitamin K_3 _2 mg; Thiamin 2 mg; Riboflavin 5 mg; Vitamin B_6 _3 mg; Vitamin B_12 _22 ug; Pantothenic acid 18 mg; Niacin 22 mg; Biotin 0.2 mg; Folate 1.5 mg.

^d^Microminerals: Providing the following amounts per kg of feed: Zn 112 mg; Fe 80 mg; Mn 60 mg; Cu 16 mg; I 1 mg; Se 0.4 mg.

### Study designs

The experimental period lasted for three weeks. Twelve pigs were randomized into two groups (n = 6) and individually fed one of two diets; CLA+VA or REG. The pigs were weighed once every week and amount of feed was adjusted according to body weight. At feeding time, pigs were restrained in an individual feeding stall for about 1/2 h, and feed intake was recorded.

Blood samples were obtained by vena cava puncture at the start and at the end of the experiment. Blood samples were taken after an overnight fasting in Na-heparin-, EDTA- and empty vacuum tubes. Blood samples were immediately chilled on ice. Plasma and serum were obtained by low speed centrifugation for 20 min at 1700 g. Plasma, serum and whole blood (heparin blood) were frozen and kept at -80°C until analyzed.

### Fatty acid analyses

Fatty acid composition in serum, feed concentrate, palm oil and butter were determined by gas chromatography. Lipids were extracted according to Folch et al. [26]. For lipid extraction from serum a modified method was used: 0.2 ml serum was mixed with 0.3 ml 0.5 M KH_2 _PO_4_, 1.5 ml chloroform and 0.5 ml methanol. After centrifugation at 1700 g for 10 minutes, the lower phase was transferred to new tubes, the solvents were evaporated by N_2_, and lipids resolved in heptane. Fat from serum and diet were methylated by the method described by Kramer et al. [27], using both sodium methoxide and methanolic HCl 3N (Supelco, PA, USA). Subsequently, the fatty acid methyl esters were analyzed using a Finnigan Focus gas chromatograph with a 100 m capillary column (CP Sil 88 WCOT, 100-m × 0.25 mm, Chrompack, Middelburg, Netherland). Peak areas of fatty acids were used to calculate the amount of fatty acids (g/100 g fat) by theoretical response factors [28]. Standard fatty acids of known composition were run to identify the fatty acids in the samples. Plasma control samples were extracted, methylated and analysed by every 10^th ^sample.

### Plasma analyses

The heparin-plasma analyses were carried out on a Cobas Mira autoanalyzer using the following kits: nonesterified fatty acids (NEFA) (NEFA C ACS-ACOD method. Wako Chemicals, VA, USA), triacylglycerol (Triglycerides 100, ABX diagnostics, Montpellier, France), total cholesterol (Cholesterol 100–250, ABX diagnostics, Montpellier, France), high density lipoprotein-cholesterol (HDL-cholesterol direct, ABX diagnostics, Montpellier, France) and glucose (Glucose HK 125 kit, ABX diagnostics, Montpellier, France). Low density lipoptotein-(LDL) cholesterol was calculated using the Friedewald equation [29]. The interassay coefficients of variation were the following: total cholesterol 2%, HDL-cholesterol 5%, triacylglycerols 3%, NEFA 2.5%.

LDL particle size distribution was determined by gradient gel electrophoresis as described by Sjogren et al. [5]. Briefly, a lipoprotein-rich fraction (containing very low density lipoprotein (VLDL) to LDL) was isolated from freshly thawed EDTA-plasma by adjusting the density to 1.070 kg/L and subsequent ultracentrifugation (142500 g for 22 h, 4°C). Recovery of total plasma apoB was 77 ± 12% (n = 8). The lipoprotein-rich fraction was applied to a 3–7.5% polyacrylamide gel together with standard lipoproteins (isolated human Lp(a) and LDL) and proteins (thyroglobulin mono- and dimer, Pharmacia, LKB, Stockholm, Sweden) of known size and run for 20 h at 80 V. Gels were stained for protein (0.04% Coomassie Brilliant Blue, Serva, Heidelberg, Germany) and analyzed using a Fuji LAS-1000 system and Image Gauge software to give peak particle size of LDL and relative distribution of LDL in predefined subfractions with cut-offs: LDL-I (27.0–25.0 nm), LDL-II (25.0–23.5 nm), LDL-III (23.5–22.5 nm) and LDL-IV (22.5–21.0 nm), corresponding to densities of 1.006–1.030, 1.030–1.040, 1.040–1.050 and 1.050–1.063 kg/L, respectively. A density of 1.040 kg/L is a classic boundary for dividing LDL into large and small particles [30] rendering LDL subclasses III and IV as small dense LDL with this method.

### Statistical methods

The results of the plasma and serum analyses are presented as mean values, and standard deviation and p-values are given. Data were analyzed by using the statistical package in Microsoft Office Excel, 2003, using TTEST, two-tailed distribution and two-sample equal variance.

## Competing interests

The authors declare that they have no competing interests.

## Authors' contributions

AH, PS, NH, NPK, OT, NF, HM, ASB, ES–O and OMH have made substantive intellectual contributions to the study concerning conception and design, acquisition of data and analyses and interpretation of the data.
